# Gene Regulatory Mechanism of Mycobacterium Tuberculosis during Dormancy

**DOI:** 10.3390/cimb46060348

**Published:** 2024-06-11

**Authors:** Yiduo Liu, Han Li, Dejia Dai, Jiakang He, Zhengmin Liang

**Affiliations:** 1 College of Animal Science and Technology, Guangxi University, No. 100 University West Road, Nanning 530004, Chinadaiuon@163.com (D.D.); 2 College of Veterinary Medicine, China Agricultural University, Beijing 100193, China

**Keywords:** latent tuberculosis infection, dormancy, regulatory mechanism, vaccines

## Abstract

Tuberculosis (TB), caused by *Mycobacterium tuberculosis* (Mtb) complex, is a zoonotic disease that remains one of the leading causes of death worldwide. Latent tuberculosis infection reactivation is a challenging obstacle to eradicating TB globally. Understanding the gene regulatory network of Mtb during dormancy is important. This review discusses up-to-date information about TB gene regulatory networks during dormancy, focusing on the regulation of lipid and energy metabolism, dormancy survival regulator (DosR), White B-like (Wbl) family, Toxin-Antitoxin (TA) systems, sigma factors, and MprAB. We outline the progress in vaccine and drug development associated with Mtb dormancy.

## 1. Introduction

Tuberculosis (TB), which a chronic infectious disease caused by *Mycobacterium tuberculosis* (Mtb), caused around 1.30 million deaths in 2022 [1]. In comparison, it is estimated that about 1.7 billion people are living with latent TB infection (LTBI) [2]. The characteristic of LTBI is that Mtb is in a dormant state, and the body does not show clinical symptoms or expel bacteria. However, persons with LTBI may progress to active TB at any time [3]. The COVID-19 pandemic has reminded us of our focus on healthcare workers (HCWs), while HCWs have a higher risk of acquiring the infection in hospital settings because of frequent close exposure to patients suffering from TB [4]. Therefore, it is essential to find strategies that can significantly reduce the likelihood of latent TB progressing to active TB for TB control [2]. Healthcare interventions may reduce the risk of progression from LTBI to active TB disease. More effective drugs for the treatment of LTBI and new effective TB vaccines for the prevention of LTBI reactivation in adults are needed to achieve The End TB Strategy set for 2030 [1]. 

**Figure 1:**
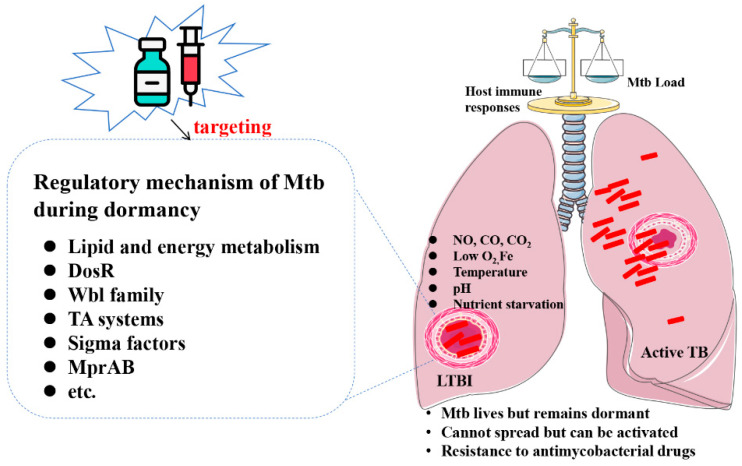
**Characteristics of latent tuberculosis infection.** Mtb is in a dormant state, and the body does not show clinical symptoms or expel bacteria. On the one hand, the host prevents mycobacterial replication via acid stress, oxidative stress, cell surface stress, hypoxia, nutrient starvation, essential-element starvation, genotoxic stress, etc. On the other hand, the dormant Mtb can evade host immune clearance by the regulation of lipid and energy metabolism, DosR, Wbl family, TA systems, sigma factors, MprAB, etc. Mtb genes associated with persistent infection are potential targets to prevent and control latent tuberculosis infection.

Most cases of TB are caused by respiratory infections and typically involve the lungs. As the condition progresses, the balance between Mtb and host immune resistance will cause the formation of granuloma structures. A complete granuloma can control the pathogen and prevent its transmission, which is beneficial to the host. During this period, according to Figure 1, the host prevents mycobacterial replication through numerous defenses, including acid stress, oxidative stress, cell surface stress, hypoxia, nutrient starvation, and genotoxic stress [5]. The ability of Mtb to resist exogenous stress factors, such as hypoxia, nitric oxide (NO), heat shock, nutrient starvation, low pH, and low iron, is critical for its persistence. These factors promote transition of Mtb to the dormant non-replicating state [6]. But this balance will be disrupted when the immune system declines, and Mtb will break through local defenses and escape, causing active TB. Therefore, it is important to understand the key factors that restrict the dormancy of TB for the control of latent TB. Mtb maintains the dormant phase from three aspects: immunity (Toll-like receptors, cytokines, and immune cell function), biochemistry (drug resistance), and genetics (activation of genes associated with dormancy) [7]. In addition, the ability of Mtb to survive acidic stress conditions in host macrophages is a key feature of persistent infection [8]. Interestingly, the DosR regulon, the WhiB-like (Wbl) proteins, the sigma (σ) factors, and the MprAB system appear to be implicated in response to stress associated with persistent Mtb infection.

The latest developments in understanding the roles of Mtb genes associated with the ability of resistance against antimicrobial molecules and adaptation to host-induced metabolic constraints in the establishment of the dormant stage and the control of TB as potential candidates for designing anti-TB drugs or vaccines are discussed in this review.

## 2. Morphological Changes in Mtb during Dormancy

**Figure 2:**
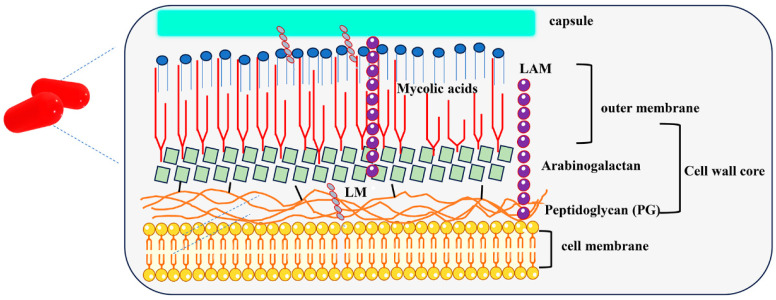
**Schematic diagram of Mtb cell wall.** The cell wall structure of Mtb comprises four main layers: the cell membrane, peptidoglycan layer, outer membrane layer, and capsule successively from the inside out. The outer layer of mycolic acids surrounds inner layers of arabinogalactan and peptidoglycan.

Dormant Mtb undergoes a series of changes in order to respond to environmental stress, such as the metabolic rate slowing down, cell volume increasing and cell membrane thickness increasing, and other functional structural changes. The length of dormant Mtb increases in hypoxic environments [9], while Mtb exhibited thickened cell walls and decreased metabolic activity in an acidified environment (pH 4.7) [10]. Transmission electron microscopy showed that Mtb in the dormant period had a rough and uneven surface [11]. Recent studies have focused on changes in the cell wall composition of Mtb during dormancy [12]. The cell wall of Mtb is mainly composed of polysaccharides and lipids with unique and atypical structures. The cell wall structure of Mtb is shown in Figure 2, with the cell membrane, peptidoglycan (PG) layer, outer membrane layer, and capsule successively from the inside out [13]. It was found that the contents of lipophilated mannan (LM) and arabinomanolipid (LAM) in the cell wall of Mtb during dormancy, and the molecular weight of LAM and the ratio of arabinose to mannose, also increased. Mtb may alter the structure of LAM by increasing the level of arabinosylation in the mannan domain in response to nutrient consumption [14]. The pe/ppe genes are found to be associated with the alteration of mycobacterial cell wall integrity. PE_PGRS were reported to thicken the bacterial cell wall to cope with external stimuli by affecting fatty acid metabolism and accelerating the synthesis of lipids such as mycotic acid [15].

## 3. Regulatory Mechanism of Mtb during Dormancy

### 3.1. Lipid and Energy Metabolism

**Figure 3:**
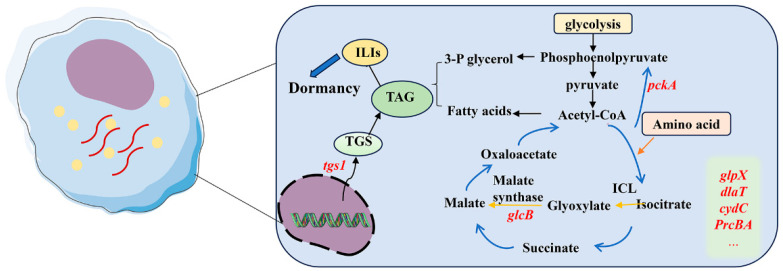
**Mechanisms of lipid and energy induced by Mtb in host cells.** Fatty acids (acetate or propionate) alter Mtb central carbon metabolism, triggering dormancy and drug tolerance. In various stresses, Mtb synthesizes triacylglycerol (TAG, encoded by tgs1) utilizing fatty acids from host to induce the formation of intracytoplasmic lipid inclusions (ILIs). Phosphoenol pyruvate and pyruvate may provide the precursors for acetyl-CoA. Isocitrate lyase and malate synthase (encoded by glcB) from the glyoxylate pathway are upregulated during dormancy. Other enzymes, including MenA, methionine aminopeptidase, fatty acid CoA synthetase, glycine dehydrogenase, and l-alanine dehydrogenase, are involved in fatty acid and amino acid biosynthesis, which are related to genes such as glpX, dlaT, cydC, and PrcBA.

The formation of latent infections requires energy and biomass. A previous study shows that Mtb requires host cholesterol to survive, and mce4 encodes a cholesterol import system that assists Mtb to acquire carbon and energy from host cholesterol [16]. The ability of Mtb to prioritize the use of lipids as its carbon source is a metabolic characteristic that can lead to chronic infections [17,18,19,20]. Fatty acids are normally stored as triacylglycerol (TAG) encoded by tgs1 (a DosR-regulated gene). Mtb acquires fatty acids from the host for the synthesis of TAG, which is subsequently stored in the form of intracytoplasmic lipid inclusions (ILIs) to satisfy the demands for carbon and nutrients, thereby limiting metabolic stress and enhancing mycobacterial dormancy [21,22,23]. Conversely, excess carbon and nitrogen starvation promote TAG accumulation as ILIs in Mtb [24]. Figure 3 summarizes the potential routes of TAG synthesis in Mtb. The glyoxylate shunt is a metabolic pathway used by bacteria to absorb linked fatty acids and has been associated with persistence of Mtb. Isocitrate lyase (ICL) isoforms 1 and 2, which are the first enzymes of the glyoxylate shunt, are involved in the glyoxylate and methylcitrate cycles and are critical to the ability of Mtb to replicate and persist in mice [22,25,26,27]. The next enzyme of the glyoxylate shunt, malate synthase encoded by glcB, is also essential for persistence during the chronic phase of mouse infection [28]. On the other hand, gluconeogenesis is important for the conversion of fatty acids into biomass, which is essential for growth of Mtb on fatty acids. Phosphoenolpyruvate carboxykinase, encoded by pckA in Mtb, catalyzes the first committed step of gluconeogenesis. Quinonez et al. determined the metabolic status of PEPCK knockout strains (ΔpckA) cultured in fatty acid or glycerol media [29]. In fatty acid media, ΔpckA failed to grow but remained viable, while it grew at levels similar to that of wildtype in glycerol media, implying that ΔpckA may use preexisting carbon sources to fuel glycolysis [29]. The Mtb fructose 1, 6-bisphosphatase encoded by glpX is a key enzyme of gluconeogenesis [30]. In addition to lipid metabolism, other metabolic pathways are involved in long-term survival of Mtb in the host. dlaT, a component of alpha-ketoacid dehydrogenase complexes which are central to intermediary metabolism, supports Mtb’s antioxidant defense [31] and participates in the constitution of pyruvate dehydrogenase [32]. menA participates in the synthesis of menaquinone which is critical for fatty acid and amino acid biosynthesis [33]. The expression of cydABDC (an annotated four-gene cluster) is increased by exposure to hypoxia or NO in vitro and during the chronic phase of Mtb infection in mice [34], while cydC is the last ORF in cydABDC, and cydC disruption is found to impair Mtb persistence in isoniazid-treated mice [35]. Conditional gene silencing [36] or genetic depletion [37] of the core proteasome subunit PrcBA caused hyper-susceptibility to reactive nitrogen intermediates and impaired bacillary long-term survival during chronic infection, suggesting that proteasomal proteolysis facilitates mycobacterial persistence. The main proteins involved in the regulation of Mtb DosR are shown in Table 1.

### 3.2. DosR 

**Figure 4:**
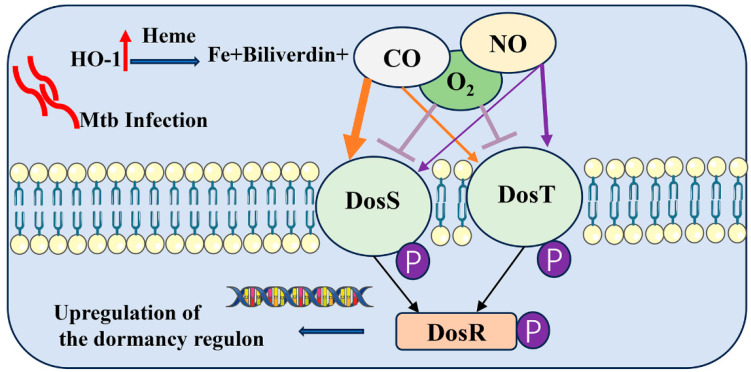
**Schematic diagram of the DosS-DosT/DosR sensor system.** Mtb responds to hypoxia through the DosR regulon, which is activated by kinases DosS and DosT. Macrophage infection by Mtb induces HO-1, which catabolizes heme to release CO, iron, and bilverdin. The deoxy ferrous form of DosS/DosT is autophosphorylated under hypoxia or upon binding CO or NO. The phosphorylated protein transfers the phosphate group to DosR, which then binds DNA, resulting in downstream signaling and leading to the upregulation of the genes necessary for dormancy.

The dual component system DosS-DosT/DosR has received significant attention over the last decade as a key regulatory mechanism for LTBI (Figure 4). Latency in Mtb is regulated by the DosR regulon, which is a set of approximately 50 genes [7]. A combined omics analysis revealed that deleting DosR significantly upregulates L-aspartic acid and serine synthesis, while downregulating seven other amino acids, including L-histidine and lysine [46]. This suggests that DosR regulates amino acid synthesis and metabolism [47]. The system containing two sensor kinases, DosS (*Rv3132c*) and DosT (*Rv2027c*), and a response regulator DosR (*Rv3133c*) assists Mtb to adapt to anaerobic conditions, thereby allowing it to survive during latent infection [48,49]. The DosR gene is activated in a microenvironment with low oxygen and elevated levels of NO, CO, and reactive oxygen species (ROS). The DosR-dependent regulon is induced in response to stress conditions, including hypoxia [6,50], NO [6], and carbon monoxide [51], suggesting the role of the DosR regulon in maintaining bacterial growth under unfavorable conditions. Moreover, it was demonstrated that the DosR regulon regulates adaptive immunity to maintain persistent infection of Mtb in response to hypoxia in a macaque model, thus regulating the Mtb life cycle and affecting lung pathology [52]. The protein products encoded by DosR genes are reported/predicted to participate in various functions, and they were classified into eight groups including stress response, host–pathogen interactions, redox balance, and so on [53,54]. The functional roles of these major DosR genes are outlined in Table 2. 

### 3.3. Wbl Family as Intracellular Redox Sensors

**Figure 5:**
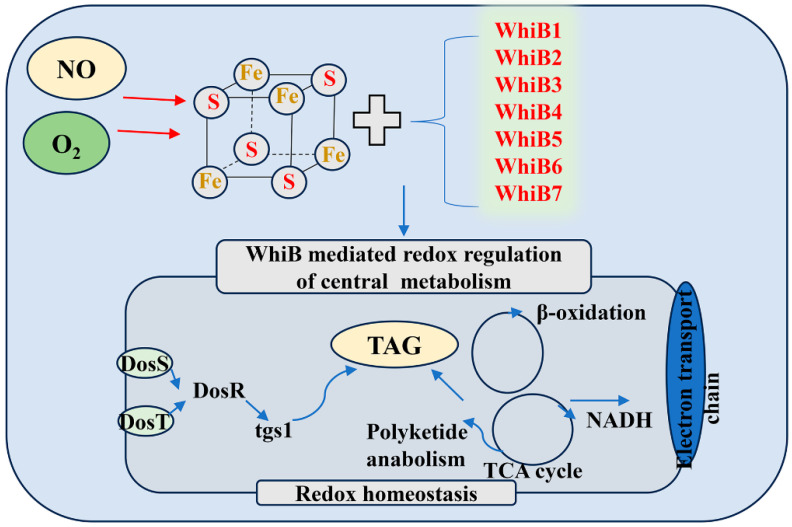
**Schematic diagram of WhiB-like (Wbl) family as intracellular redox sensors.** Seven Wbl proteins, as intracellular redox sensors, share about 30–50% sequence identity, and they regulate DosR regulons through their [4Fe–4S] cluster in response to the dormancy signals NO and O_2_ and are involved in maintaining redox homeostasis via regulating triacylglycerol (TAG) metabolism.

It is worth mentioning that the proteins of the Wbl family have been shown to exhibit important pleiotropic roles, including response to the dormancy signals NO and O_2_ [82,83,84,85], as shown in Figure 5. In Mtb, seven Wbl proteins (WhiB1–7) have been identified and are associated with developmental processes in Mtb [82]. The whiB1 gene is essential, and its protein product, a DNA-binding protein, possesses a NO-sensitive iron–sulfur cluster [4Fe–4S] holding four-helix bundles with a core of three α-helices [83,84]. The iron–sulfur cluster reacts with eight molecules of NO [84]. WhiB3, which represents the prevailing paradigm among Wbl proteins, has an [4Fe–4S] cluster which specifically reacts with O_2_ and NO and other metabolic signals to maintain redox balance [85]. Expression of WhiB3 is induced in the presence of hypoxia, NO [86], and ROS in macrophages [87]. It was demonstrated by a paradigmatic mechanism that WhiB3 maintains intrabacterial redox balance by regulating Mtb lipid anabolism [88]. Moreover, WhiB3 expression is also increased under acidic conditions in vitro [89] and inside macrophages [90]. A recent study has shown that this pH-dependent upregulation in WhiB3 expression is mediated by the PhoPR regulon [91]. In addition, WhiB3 is regarded as a crucial mediator of phagosomal maturation arrest and acid resistance as Mtb regulates phagosomal pH by downregulating the expression of innate immune genes [92]. Moreover, WhiB3 regulates the expression of virulence genes involved in blocking phagosomal maturation (e.g., polyketides, RD-1 antigens), which allows Mtb to thrive in growth-permissive environmental conditions [93]. Additionally, WhiB3 also induces the expression of several genes involved in maintaining redox homeostasis and protection from ROS and RNS. Like other members of the Wbl family, WhiB4 contains an O_2_- and NO-sensitive [4Fe–4S] cluster and controls the oxidative stress response in Mtb [94]. Interestingly, a recent study has shown that WhiB4 modulates redox homeostasis by both directly and indirectly affecting the expression of genes in response to oxidative stress [95]. Furthermore, a study on a zebrafish model infected with *Mycobacterium marinum* reported that WhiB6 differentially regulates the ESX-1 and DosR regulons through its [4Fe–4S] cluster in response to NO or O_2_ to establish sustained infection and maintain the integrity of granulomas [96]. In common with most other Wbl proteins, WhiB7 regulates thiol redox balance in Mtb [97] and acts as a redox-sensitive transcriptional activator in Mycobacterium smegmatis [98]. The function of Wbl family proteins is shown in Table 3.

### 3.4. TA Systems

**Figure 6:**
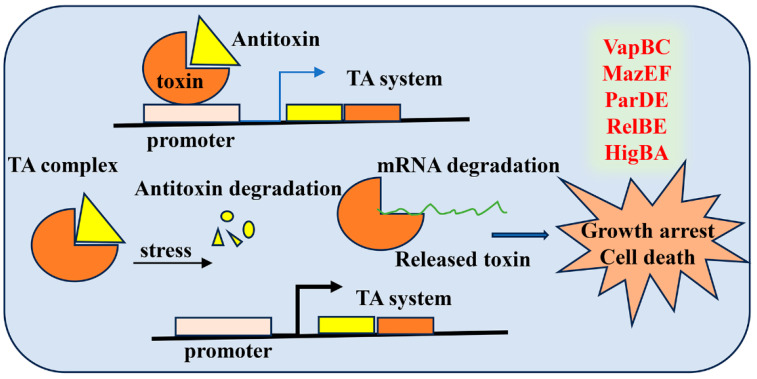
**Schematic diagram of the regulation of TA systems.** TA systems are generally centered at a promoter region, which are cotranscribed and are mostly cotranslated as a stable toxin and a relatively unstable antitoxin together, forming the TA complex. In response to environmental stresses, the antitoxin is degraded and leads to toxin activation. The toxin proteins are activated and degrade mRNA, resulting in cell death.

Toxin–antitoxin (TA) systems are widely present in many bacterial genomes, including that of Mtb. TA systems are composed of two component genetic modules comprising adjacent genes encoding two small proteins, which are the toxin and its homologous antitoxin. The TA systems play a significant role in growth, antibiotic tolerance, and persistence of bacteria [101]. Mtb possesses approximately 90 TA systems in its chromosome, and some of them are strongly induced in drug-tolerant persisters [102,103]. TA systems are thought to be involved in the survival of Mtb under stress and the establishment of LTBI, as they typically impart reversible growth inhibition upon stresses associated with this state [104,105]. Mtb is subjected to stresses relevant to LTBI, such as hypoxia [106,107], nutrient limitation [106,108], macrophage infection [107,109], antibiotic treatment [106,110,111], and increased expression of TA toxins. A recent study shows that toxin-mediated proteome reprogramming may facilitate Mtb stress survival via tandem with other pathways [103]. The cumulative results indicate that TA systems participate in regulating adaptive responses to stresses associated with the metabolic restriction environment in the host. Currently, six TA families based on the mode of inhibition of the toxin by the antitoxins have been reported [101]. However, TA type II, including TA families VapBC, MazEF, ParDE, RelBE, and HigBA, is mainly present in MTBC strains [102,105], as shown in Figure 6. 

Several modules belonging to the largest TA family VapBC were found to be upregulated within LTBI granulomas of a non-human primate model [112]. MazEF TA family members (MazF3, MazF6, and MazF9) synergistically promote Mtb’s ability to adapt to oxidative stress, nutrient depletion, and drug exposure [106]. The TA genes of the RelBE family inhibit mycobacterial growth via translation inhibition and are highly expressed under nitrogen-limiting and oxidative stress conditions [109,113]. However, the deletion of each of the RelE toxins does not affect survival during Mtb infection in mice, suggesting that these toxins may not individually contribute to the antibiotic tolerance of Mtb in vivo [111]. Three higBA loci of the HigBA family in Mtb are highly expressed under chemical and environmental stress conditions [114]. Altogether, these data support the idea that TA systems play an important role in the establishment and maintenance of LTBI and persistence of Mtb. 

Multiple TA proteins cooperatively function in response to changing environmental conditions. However, the interaction between different TA systems and the difference in TA regulation between different mycobacterial lineages are unclear. In vitro studies have shown that levels of multi-drug tolerance significantly reduced only after sequential deletion of at least five homologous type II toxins, suggesting functional redundancy.

### 3.5. Sigma Factors

It is worth mentioning that a class of transcription factors, sigma (σ) factors, allow Mycobacterium to successfully adapt to stress conditions. Mtb has 13 different putative sigma factors [115]. The housekeeping sigma factor directs RNA polymerase (RNAP) to genes required for basic functions in normal growth environments, while the assistive sigma factor reprograms RNAP to transcribe genes involved in stress responses [115,116]. The principal σ factor SigA, with a housekeeping function, was reported to be capable of interaction with WhiB1 [84], WhiB3 [117], and WhiB7 [98]. SigB transcription is increased during the stationary phase of Mtb, and it controls the regulons of stationary phase and general stress resistance [118]. In addition, SigD is increased during nutrient starvation [119], indicating the function of SigD in physiological adaptations such as stringent response and starvation. It has been demonstrated that RpfC associated with the revival of dormant mycobacteria is among the most strongly regulated genetic targets of SigD [120,121]. Like other sigma factors, SigE is involved in hypoxia-induced dormancy by regulating central metabolic genes (icl1 and gltA1) [122], which is essential for survival under different stressful conditions [123]. Moreover, a recent SigE mutant study showed that SigE is not directly involved in the activation of stringent response but in protecting the cell from metabolic stress associated with adaptation to low phosphate and activation of stringent response [124]. In common with most other sigma factors, SigH is also an alternative sigma factor which is involved in response to oxidative and nitrosative stress and heat shock [125]. Moreover, a murine study has shown that SigH is required for the production of immunopathology and mortality [126]. Moreover, SigF mRNA is strongly upregulated under the conditions of stationary phase, nitrogen depletion, and cold shock, suggesting it may play a role in the adaption to host defenses and persistent Mtb infection [127]. In addition, SigE and SigB are involved in Mtb’s tolerance to antitubercular drugs and persistence, suggesting the possibility of developing drugs that target specific sigma factors [128]. The proteins involved in the posttranslational regulation of Mtb sigma factors are shown in Table 4.

Regulator of SigE A (RseA), regulator of Sig A (RshA), and regulator of Sig L A (RslA) belong to the ZAS family of proteins were identified. Two cotranscribed genes exist downstream of Mtb rseA, the first encodes a membrane serine protease (HtrA), and the second encodes a twin-arginine translocation (Tat) system protein, TatB. Interestingly, the RseA N-terminus region has two arginine residues, suggesting that this protein could be targeted to the bacterial membrane through the Tat system to allow its degradation by HtrA [115]. However, there was no clear evidence to demonstrate interactions between these proteins.

### 3.6. MprAB

In addition to the above-mentioned genes implicated in stress response during LTBI, the genes involved in the typical two-component regulatory systems (TCSs) are important for adaptation of Mtb under stress conditions [133,134]. Especially, the MprAB system contains sensor kinase MprB and response regulator MprA, as part of a cell envelope stress response network, regulating resistance to cell envelope stress [135,136,137]. A study has shown that TCSs are required for the formation and maintenance of latent Mtb infections in mice [138]. It has been demonstrated that MprAB-mediated signal transduction is negatively regulated by the extracellular domain (ECD) of MprB. Under non-stress conditions, the signaling via the MprAB system is inhibited by the MprB ECD, while it is removed and subsequently activated under cell envelope stress conditions [137]. In addition, MprAB regulates the expression of many stress response genes, such as SigE and SigB [135], and regulates the function of ESX-1 [139]. 

### 3.7. Other Genes Associated with Dormancy

*Rv2660c* is the most upregulated gene in a model of Mtb starvation, which is crucial for Mtb to adapt to nutrient-deficient and hypoxic environments [119]. PhoY1 and PhoY2 can promote persister formation by mediating phosphate-sensing signals [140]. Rpfs promote survival of Mtb in vivo [141], associated with protection in long-term Mtb-infected individuals [142]. Rel mediates ppGpp hydrolysis [143], which can help Mtb entry into quiescence [144], and is therefore a promising drug target. PPK1 is responsible for inorganic polyphosphate (PolyP) synthesis [145]. PPK2 is required for Mtb PolyP regulation and virulence [146,147,148]. PPX1 and PPX2 modulate PolyP degradation, contributing to the ability of Mtb to survive in nutrient-limiting, low-oxygen growth conditions [149]. A recent study found that *Rv1255c*, a dormancy-related transcriptional regulator of the TetR family in Mtb, promoted isoniazid tolerance in Mycobacterium smegmatis via orchestrating drug efflux machinery [150]. A recent study showed that the melH gene in Mtb and *Mycobacterium marinum* was vital to maintain a delicate equilibrium of redox, and deletion of melH led to increased sensitivity to oxidative stress [151].

### 3.8. Temporal Regulation of Key Genes and Proteins during Dormancy

Dormancy of Mtb within macrophages, a key virulence trait of Mtb, requires dynamic adaptation to diverse and changing conditions. Studies have examined gene transcription and expression during TB infection in macrophages by transcriptomic or proteomic methods. A study of a 14-day macrophage model infected with Mtb showed that gene expression changes of maximal magnitude and complexity occurred at day 2 [152]. Most of these genes are regulated in immediate response to the stressful conditions, including genes involved in general stress responses (hsp, dnaK, groEL2), stress response regulation (SigB), anaerobic respiration (type II NADH dehydrogenase, ndh2), Fe-S cluster assembly (*Rv1460*, *Rv1461*, *Rv1465*), regulation of acetate and glyoxylate cycle metabolism (*Rv0465c*), and propionate metabolism (*Rv1129c*, prpC (*Rv1131*), prpD (*Rv1130*)). The temporal changes in Mtb gene expression during the intracellular adaptation phase (following day 2) will indicate the metabolic pathways and survival mechanisms required for establishment of an environment supportive to bacterial growth. Research showed 103 genes induced early (maximal by day 2 p.i.) that remain elevated throughout the 14-day time course, including genes involved in fatty acid metabolism (pckA, icl, ald, *Rv0448c-0449c*), KstR-dependent cholesterol metabolism (igr), secreted antigens (*mpt83-Rv2874-mpt70*), and regulators (*Rv2359*). The most striking aspect of dormancy gene expression during long-term infection was the sudden repression of DosR-dependent genes on day 8, despite steady transcript levels of DosR, DosS, and DosT. Evidence suggests that regeneration of dormant Mtb begins with resuscitation. So-called resuscitation-promoting factors (Rpfs) have been shown to induce resuscitation, and Mtb comprises five Rpf genes [153].

The proteomic results showed the DosR regulon makes up 20% of the total protein biomass during dormancy [154]. Other strongly (>4-fold) induced proteins include alanine dehydrogenase (Ald), several proteins involved in lipid metabolism (FadE5, DesA1/2, Tgs1/4, and Icl1), and the copper stress-related enzymes MymT (copper toxicity protection) and CsoR (copper-sensitive operon repressor). Proteins that responded transiently within 6 h to reaeration but returned to preaeration levels within 2 days include PrpC and PrpD, two enzymes of the methylcitrate cycle, as well as the sigma factors SigE and SigB and the transcriptional regulator ClgR, suggesting their involvement during resuscitation.

## 4. TB Vaccines Based on Antigens Associated with Dormancy

The live attenuated Bacille–Calmette–Guérin (BCG) TB vaccine, the only licensed vaccine against TB, provides consistent protection against severe forms of childhood TB [155]. However, BCG-elicited immunity loses its potency gradually, leading to insufficient protection [156], and fails to prevent reactivation disease as a result of LTBI [157]. Therefore, there is an urgent need to develop new vaccines that have sufficient efficacy to replace BCG or enhance the long-term efficacy of BCG in preventing TB.

Most current vaccine candidates suffer from inability to eliminate LTBI, and the majority of TB vaccine candidates currently in clinical trials were based on antigens expressed in replicating stages [158]. These TB vaccines can prevent active TB infection but not LTBI. According to the current literature, a TB vaccine should contain latent phase antigens [159,160,161]. Knowledge of regulation of key genes and proteins during dormancy might also assist in the development of treatments and preventive measures to fight TB. DosR is a key mediator in Mtb adaptation to dormancy in response to gaseous stresses such as hypoxia, carbon monoxide, and NO [6,162,163]. The protein products of many DosR regulon genes appear to be good antigens and involved in bacteria–host interactions. Therefore, it is considered as a novel target for developing strategies against dormant bacteria [162,164,165,166]. *Rv2623* [56], *Rv2031c* [167], and *Rv2626c* [168] are potential biomarkers for diagnosis. *Rv2031c* is also a well-characterized member of the DosR regulon and may stabilize cellular structure during hypoxia [169]. It is an important antigen which can induce specific humoral and cellular immune responses in individuals with LTBI [170,171]. A subunit vaccine incorporating *Rv2031c* [172] and BCG over-expressing Rv2031c [173] are potential vaccine candidates. Our previous study showed that Rv2031c combined with Ag85A induced effective immune response against *Mycobacterium bovis* [174]. *Rv2660c* (as a component of H56) [175], *Rv1813* (as a component of ID93) [176], *Rv1735c* [64], *Rv1733c* [66,67], *Rv2627*, and *Rv2628* [177] are also promising candidates for incorporation into subunit vaccines against TB. Currently, multi-stage TB vaccines which include the multi-component antigens from “early secreted antigens” and “latency antigens” are increasingly focused upon. Among these multi-stage vaccines, H56 and ID93, in combination with adjuvants, have entered clinical trials [175,176]. In 2018, the TB vaccine candidate M72/AS01E was found to be significantly protective against TB in individuals with LTBI in a Phase IIb trial [178].

## 5. Drugs Associated with Dormancy

The treatment of TB is mainly dependent on drug chemotherapy. During past decades, rifampicin (RIF), isoniazid (INH), ethambutol (EMB), and pyrazinamide (PZA) have been classified as the first-line anti-TB drugs, and the second-line drugs include fluoroquinolones, amikacin, and kanamycin. However, dormant non-replicating persistent (NRP) Mtb is prone to develop resistance to these anti-TB drugs, leading to the inability to effectively clear NRP Mtb, thus prolonging the treatment cycle of TB. The key to the treatment of TB is to prevent LTBI from developing into active TB. Anti-TB drugs targeting dormant Mtb act mainly by inhibiting the metabolic process of the pathogen and the detoxification or repair pathways of Mtb itself [179]. In recent years, many new drug treatment plans have been approved for the clinical prevention and treatment of LTBI. The combination protocol of INH with rifamycin (RIF) [180] and rifapentine (RFP) [181] plays a significant role in the treatment of LTBI. PZA has a strong bactericidal activity against semi-dormant mycobacteria [182]. For murine LTBI, the bicyclic nitroimidazole-like molecule PA-824 alone has a similar efficacy to INH alone [183] and combined with sutezolid provides an effective treatment of LTBI in MDR-TB [184]. The National Tuberculosis Controllers Association and CDC updated treatment guidelines for LTBI in 2020 are: weekly INH plus RFP for three months, daily RIF for four months or daily INH plus RIF for three months, and the alternative regimen is daily INH for six or nine months [185]. Due to the increasing resistance of LTBI to the above drugs, the development of new drugs and improved treatment options is imminent, and there are many candidates against LTBI in clinical trials, such as sutezolamide, delpazolid, telacebec, OPC-167832, SQ 109 [186], tafenoquine [187], and so on. Notably, *Rv0467*, encoding isocitrate lyase (ICL), is a drug target for the treatment of latent TB, but there is no ICL inhibitor that has reached clinical trials [188]. Moreover, novel inhibitors targeting PcaA are expected to control the dormant state of Mtb [43]. Recently, melH [151] showed potentiality as an attractive target for the development of novel antitubercular therapies.

However, new anti-TB compounds are targeted at blocking the enzymes that remain active during the dormant state of Mtb, including alanine dehydrogenase, isocitrate lyase, cysteine synthase CysM, adenosine-5′-phosphosulfate reductase, DevS and DosT oxygen sensors involved in dormancy response, decaprenyl-phosphoryl-ribose 2′-epimerase (DprE1), mycocyclosin synthase (Cyp121), and extracellular zinc metalloprotease 1 (Zmp1) [189]. 

Another possible direction of the anti-TB drug design is preventing TB reactivation via inhibiting Rpfs, which are hydrolases with muralytic activity that are able to cleave the cell wall of dormant Mtb, thus preventing their resuscitation [190].

## 6. Concluding Remarks

The novel coronavirus pandemic was a major turning point, with many targets missing, and patients infected with Mtb received significantly less treatment, which recovered in 2022. The World Health Organization (WHO) emphasized the role of new TB vaccines in the prevention and control of TB and called for approving at least one new vaccine until 2027. The development of new vaccines needs to include finding new and reliable targets. The WHO recommends systematic surveillance of at-risk populations, including household contacts of TB patients, people living with human immunodeficiency virus (HIV), and healthcare workers. Healthcare workers play a central role in eliminating TB globally, but the risk of hospital-acquired TB undermines their contribution. Early prevention of LTB reactivation, especially for people at increased risk of transitivity from LTB to active disease, such as HIV/acquired immune deficiency syndrome (AIDS) patients and those receiving immunosuppressive therapy, and HCWs, is the most important thing. The regulatory mechanisms and related genes of dormant bacilli reviewed here may be candidate targets for anti-LTBI drugs in the future. The key to fighting TB is fighting LTBI, and the existing detection means cannot fully detect LTBI, so the prevalence of LTBI is far higher than the current statistics. Once LTBI develops into active TB, it will pose a serious threat to human health. The development of new vaccines, drugs, and treatment options against LTBI is currently a top priority against TB.

During LTBI, Mtb alters its genetic program, and the antigens expressed by dormant pathogens prevail. To maintain effective control of dormant Mtb, vaccine-induced immune responses need to target antigens expressed by dormant bacilli, the so-called latency antigens. This strategy becomes more effective for postexposure vaccines given during LTBI. Therefore, a better understanding of the life cycle of TB bacteria will help rationalize the design of next-generation vaccine candidates.

## Figures and Tables

**Table 1:** Function of proteins encoded by Mtb genes participating in lipid and energy metabolism during dormancy.

Gene Name	Reported/Predicted Function of the Encoded Protein
*Rv3130c (tgs1)*	Mediates the formation of TAG [21], reduces growth and sensitivity to antituberculosis drugs [38,39].
*Rv0467 (icl)*	Encodes isocitrate lyase and facilitates the persistence of Mtb [22], involved in antibiotic tolerance [40], mycobacterial glyoxylate and methylisocitrate cycles [41].
*Rv1837c (GlcB)*	Encodes malate synthase which is essential for Mtb survival during the acute and chronic phases of infection [28].
*Rv0211 (pckA)*	Encodes phosphoenolpyruvate carboxykinase, catalyzing the first committed step of gluconeogenesis which is critical for Mtb to establish and maintain infection [42].
*Rv1099c (glpX)*	Encodes fructose 1, 6-bisphosphatase which is a key gluconeogenic enzyme for growth and survival of Mtb [30].
*Rv3499c (mce4)*	Encodes a cholesterol import system which helps Mtb to acquire carbon and energy from host membranes [16].
*Rv0470c (pcaA)*	A mycolic acid cyclopropane synthetase, associated with persistence and virulence of Mtb [43].
*Rv2215 (dlaT)*	Participates in the constitution of pyruvate dehydrogenase [32].
*Rv0534c (menA)*	Involved in the synthesis of menaquinone which is critical for maintaining mycobacterial viability [33].
*Rv1620c (cydC)*	Contributes to the generation of Mtb persistence [35].
*Rv2109c (PrcA)*	Encodes a gated proteasome, phosphorylation of proteasome affects the proteasome complex formation contributing to the survival of Mtb [44], protects Mycobacteria from antimicrobial antifolates [45].
*Rv2110c (PrcB)*	Encodes a gated proteasome, protects Mycobacteria from antimicrobial antifolates [45].

**Table 2:** Function of proteins encoded by DosR regulon.

Gene Name	Reported/Predicted Function of the Encoded Protein
*Rv2623 (TB31.7)*	Regulates mycobacterial growth by ATP-binding activity [55], acts as a biomarker for the diagnosis of latent and active tuberculous meningitis [56].
*Rv3134c*	Mediates the expression of DosR target genes [57].
*Rv2031c (HspX)*	Helps Mtb adapt to low-oxygen conditions, inhibits the maturation and differentiation of dendritic cells [58], is a TB vaccine candidate antigen [59].
*Rv2624c*	Enhances intracellular survival by ATP binding in Mycobacteria [60].
*Rv2004c*	Involved in partial drug resistance, intracellular survival, and adaptation of bacilli to stress conditions [61].
*Rv2626c*	Highly expressed under conditions of hypoxia or NO-induced stress [62], modulates macrophage effector functions, is a TB vaccine candidate antigen [63].
*Rv1735c*	Possesses immunogenicity, is a TB vaccine candidate antigen [64].
*Rv1733c*	A pathogenic antigen, highly expressed during LTBI, controls the balance between nitrate and nitrite [65], is a TB vaccine candidate antigen [66,67].
*Rv3132c (DosS), Rv2027c (DosT), Rv3133c (DosR)*	Regulates DosR regulon, encodes sensor kinases involved in the Mtb genetic response to hypoxia and NO via their autophosphorylation and phospho-transfer properties [68].
*Rv0079*	A dormancy-associated translation inhibitor (DATIN) [69].
*Rv0574c*	Modulates poly-α-l-glutamine content in the cell wall to maintain cell integrity in a hostile host environment [70].
*Rv1738*	Contributes to the shutdown of ribosomal protein synthesis during the onset of non-replicating persistence of Mtb [71].
*Rv2032 (acg)*	A nitroreductase [72], is a TB vaccine candidate antigen [73,74].
*Rv0573c (pncB2)*	Plays a role in cofactor salvage involved in biosynthesis and recycling of nicotinamide [75].
*Rv1812c*	Associated with Mtb virulence [76].
*Rv3130c (tgs1)*	Involved in accumulation of TAG under stress [77].
*Rv0569*	Contributes to signaling transduction in hypoxic conditions [78].
*Rv2030c*	Is dispensable for virulence and growth [79].
*Rv1813c*	Associated with Mtb virulence [76], manipulates the host metabolism by targeting mitochondria [80], is a TB vaccine candidate antigen [81].

**Table 3:** Function of proteins encoded by WhiB-like genes.

Gene Name	Reported/Predicted Function of the Encoded Protein
*Rv3219 (WhiB1)*	A NO-responsive transcription factor [83,84].
*Rv3416 (WhiB3)*	Responds to the dormancy signals NO and O_2_ [85] and maintains redox homeostasis and survival [93], modulates phagosome maturation and virulence [92], and interacts with the principal sigma factor [99].
*Rv3681c (WhiB4)*	An intracellular redox sensor, controls the oxidative stress response in Mtb [94,95].
*Rv0022c (WhiB5)*	A transcriptional regulator, associated with virulence and reactivation [100].
*Rv3862c (WhiB6)*	A redox-sensitive transcriptional activator of ESX-1 genes, regulates the ESX-1 and DosR regulons to modulate granuloma formation and virulence in zebrafish [96].
*Rv3197A (WhiB7)*	A transcriptional activator, regulates thiol redox balance [97].

**Table 4:** Function of proteins encoded by Sigma factors.

Gene Name	Reported/Predicted Function of the Encoded Protein
*Rv2703 (SigA)*	Promotes attachment of the RNA polymerase to specific initiation sites, cotranscribes essential housekeeping genes [129].
*Rv2710 (SigB)*	Controls the regulons of stationary phase of Mtb [118], involved in tolerance to antitubercular drugs and persistence [128].
*Rv2069 (SigC)*	Prevents copper starvation [130].
*Rv1221 (SigE)*	Involved in tolerance to antitubercular drugs and persistence [128].
*Rv3223c (SigH)*	Responds to oxidative, nitrosative, and heat stresses in Mtb [125].
*Rv3414c (SigD)*	Regulates RpfC associated with the revival of dormant mycobacteria [120,121].
*Rv3286c (SigF)*	Helps Mtb to adapt to host defenses and persist [127].
*Rv0182 (SigG)*	Involved in the SOS response in Mtb [131].
*Rv1189 (SigI)*	Interferes in SigI-RNAP interaction [132].
*Rv3328c (SigJ)*	Controls sensitivity of the bacterium to hydrogen peroxide [115].
*Rv0445 (SigK)*	Positively regulates the expression of two antigenic proteins, MPB70 and MPB83 [115].
